# Morphological characteristics influencing the orthodontic extraction strategies for Angle's class II division 1 malocclusions

**DOI:** 10.1186/s40510-014-0044-y

**Published:** 2014-07-09

**Authors:** Yongwen Guo, Xianglong Han, Hui Xu, Dongqing Ai, Huan Zeng, Ding Bai

**Affiliations:** State Key Laboratory of Oral Diseases, Department of Orthodontics, West China School of Stomatology, Sichuan University, No.14, 3rd Section, Renmin South Road, Chengdu, 610041 China

**Keywords:** Angle's class II division 1 malocclusion, Morphologic characteristics, Extraction treatment

## Abstract

**Background:**

Extraction has now been accepted widely in various malocclusions including Angle's class II division 1. However, the levels of scientific evidence in orthodontic treatment planning have been weak, and it is unlikely to systematically provide a rationale and consistent basis in decisions of extraction. This study was retrospectively designed to investigate the initial morphologic characteristics of class II division 1 subjects involving four different extraction strategies, to determine the relevant influential factors when choosing extraction strategies with the most commonly used mechanics and the principle of simplicity in orthodontic treatment based on cases diagnosed and treated by an experienced orthodontist.

**Methods:**

One hundred and ten samples of Angle's class II division 1 malocclusion with good facial and occlusal outcomes after orthodontic treatment were selected and divided into four groups according to different extraction patterns. For each case, pretreatment models and the lateral radiographs were analyzed. Significant variables of models and craniofacial structures of each group were identified by comparing the measurements using one-way analysis of variance (ANOVA) at a significance level of *P* < 0.05. Then, binary logistic regression analysis was used and a regression equation was established to quantify the correlations among the significant variables and their contributions to the extraction decisions.

**Results:**

Molar relationship, lower anterior crowding, anterior Bolton index, and anterior overjet measured from models, as well as ANS-Xi-Pm, NBa-PtGn, Li-NsPog', U1-NPog and L1-NPog measured from lateral radiographs were found to be statistically significant. Binary logistic regression analysis revealed that lower anterior crowding, molar relationship, and growth pattern were the three most relevant influential factors with a declining impact contributing to the extraction decisions for Angle's class II division 1 malocclusions.

**Conclusions:**

Angle's class II division 1 malocclusions exhibit various morphological characteristics. Orthodontists should comprehensively consider the reciprocal impact of multiple factors when choosing different extraction strategies for Angle's class II division 1 malocclusions.

**Electronic supplementary material:**

The online version of this article (doi:10.1186/s40510-014-0044-y) contains supplementary material, which is available to authorized users.

## Background

Extraction strategies have been widely accepted in various malocclusion treatments. However, disputes about teeth extraction indications in orthodontic treatment have been continued for over a century [[1]–[3]]. Appropriate extraction decisions and well-designed strategies will no doubt benefit patients and orthodontists in achieving both facial esthetics and stable occlusion. Regarding treatment for Angle's class II division 1 malocclusions, various extraction decisions exist including extractions of four first premolars, two maxillary first premolars plus two mandibular second premolars, two maxillary premolars [[4]–[6]], two maxillary premolars plus one mandibular incisor [[7]]. In rare cases, special extraction strategies, for example the extraction of molars, were used [[8],[9]]. To date, the level of scientific evidence in orthodontic treatment planning is still weak. Little evidence or criteria are available on extraction decisions for Angle's class II division 1 malocclusions with the most commonly used mechanics and the principle of simplicity in orthodontic treatment. So, it is unlikely to provide a rationale and consistent basis in decisions of extraction [[10],[11]]. A comprehensive extraction strategy should be based on considerations including but not limited to personal growth pattern, soft tissue profile, degree of crowding, molar relationship, and mid-line [[12]]. However, there is a lack of guideline or potent evidence to dictate clinical practice. Most extraction decisions are made according to personal experiences and preferences [[13]–[15]].

Therefore, this study was retrospectively designed to provide scientific evidence for extraction decisions in Angle's class II division 1 malocclusions. We collected 110 Angle's class II division 1 malocclusion cases treated by four different extraction strategies, compared the initial morphologic characteristics of subjects diagnosed and treated by an experienced practitioner with the principle of simplicity involving four different kinds of extraction strategies, and analyzed the correlation of these characteristics.

## Methods

### Sample selection

The sample was retrospectively selected from the patient files of the Orthodontic Department in West China Hospital of Stomatology, Sichuan University. In order to standardize the sample regarding treatment mechanics, the subjects were chosen from cases diagnosed and treated by one experienced orthodontist from 2008 to 2012 with pre-angulated fixed appliances (0.022 × 0.028 in.). The total number of class II division 1 cases treated with extraction was over 200. Among these, 110 cases which achieved good treatment outcomes and consisted of pretreatment and posttreatment records as well as other details of the treatment history were selected for present study. All cases were evaluated in terms of occlusal and esthetic outcomes before being included. A good occlusal result was based on a subjective evaluation of intercuspation, crown angulation, and inclination, rotations, contacts, occlusal plane, incisor, and molar relationships according to Andrew's six keys to normal occlusion. Specifically, the complete class II molar relationship with class I canine relationship was also regarded as a good outcome. What is more, in order to avoid the variable acceptability of facial esthetics from person to person, we had six orthodontic students to score the improvement of facial profiles after treatment compared to pretreatment counterparts with the help of the 100-mm visual analogue scales. Cases with an average score greater than 70 were included. Cases with dentition spaces, severe skeletal discrepancy and premolar extractions because of large-area caries or extremely ectopic position were not included. No posterior anchorage enhancement appliance (e.g., temporary implant anchorage or transpalatal arch) was used during treatment of these cases. Intra-arch elastics were applied to close the space while short-term class II elastics were used to adjust the intermaxillary relations when 0.018 × 0.025 in. stainless steel working archwires were fully engaged. The average age of patients before treatment was 14.4 years with a range of 11.7 to 17.4 years, and the average duration of treatment was 2.3 years with a range of 1.6 to 3.1 years. Samples were divided into four groups according to the extraction strategies, 30 each, except for group 2 which included 20. In group 1, two maxillary first premolars were extracted (4/), and in group 2, two maxillary first premolars and one mandibular incisor were extracted (4/1). In group 3, four first premolars were extracted (4/4) while two maxillary first premolars and two mandibular second premolars were extracted in group 4 (4/5) (Table 1).

**Table 1:** **Population sample**

Group	Extraction strategy	Number of cases
1	4/	30
2	4/1	20
3	4/4	30
4	4/5	30

### Study cast and lateral radiograph measurements

**Figure 1:**
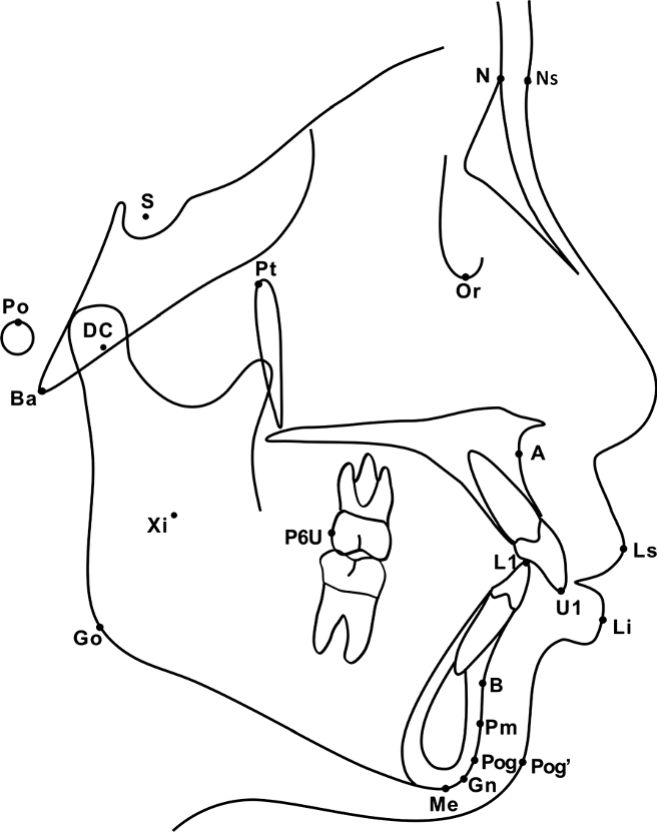
**Cephalometric landmarks used in lateral tracings.** S, sella turcica; N, nasion; Po, porion; Ba, basion; Ns, soft-tissue nasion; Pt, pterygomaxillare; Or, orbitale; A, subspinale; U1, tip of maxillary central incisor; P6U, distal point of upper first molar; L1, tip of mandibular central incisor; B, supramentale; Pm, protuberantia menti; Pog, pogonion; Me, menton; Gn, gnathion; Go, gonion; Xi, anatomical central point of mandible ramus; DC, center of condyle on N-Ba; Ls, labrale superius; Li, labrale inferius; Pog', soft-tissue pogonion.

For each case, pretreatment models were evaluated and the lateral radiographs were traced for further analysis. As shown in Table 2, anterior overjet and overbite, molar relationship, lower anterior crowding, degree of Spee's curve and Bolton index were measured from each model. Cephalometric measurements included angles of SNA, SNB, ANB, NBa-PtGn, ANS-Xi-Pm, DC-Xi-Pm, SN-GoMe, and distances of L1-APog, P6U-PTV, U1-NPog, L1-NPog, Li-NsPog', Ls-NsPog' (Table 3). Cephalometric landmarks involved in this study were shown in Figure 1.

**Table 2:** **Study cast measurements**

Number	Measurements	Definition
1	Spee's curve depth (mm)	Perpendicular distance from the line joining the mesial contact points of the lower first molars to the contact point of the lower central incisors
2	Overbite (mm)	Distance from the upper central incisor tip to the lower central incisor tip and perpendicular to the occlusal plane
3	Overjet (mm)	Distance from the upper central incisor tip to a plane tangential to the lower incisor labial surface and parallel to the occlusal plane
4	Molar relationship (mm)	Distance between the mesiobuccal cusp tip of the upper first molar and the buccal groove of the lower first molar
5	Lower anterior crowding (mm)	Discrepancy between arch length and tooth size and calculated by subtracting the arch length between distal contact points of the lower canines from the total width of lower anterior teeth
6	Bolton index 3-3 (%)	The percentage ratio of total lower anterior teeth width to total upper anterior teeth width
7	Bolton index 6-6 (%)	The percentage ratio of total lower teeth width of 6-6 to total upper teeth width of 6-6

**Table 3:** **Lateral radiograph measurements**

Number	Measurement	Definition
1	SNA°	Angle formed by the intersection of NS and NA lines
2	SNB°	Angle formed by the intersection of NS and NB lines
3	ANB°	Angle formed by the intersection of NA and NB lines
4	NBa-PtGn°	Posteroinferior angle formed by the intersection of NBa and PtGn lines
5	ANS-Xi-Pm°	Angle formed by the intersection of Xi-ANS and Xi-Pm lines
6	MP-SN°	Anteroinferior angle formed by the intersection of MP and SN planes
7	DC-Xi-Pm°	Anterosuperior angle formed by the intersection of Xi-DC and Xi-Pm lines
8	L1-APog	Distance from point L1 to line APog
9	A-NPog	Distance from point A to line NPog
10	P6U-PTV	Perpendicular distance between point P6U and line perpendicular to Frankfurt plane (Po-Or plane), tangent to Pt
11	U1-NPog	Distance from point U1 to line NPog
12	L1-NPog	Distance from point L1 to line NPog
13	Ls-Ns Pog'	Distance from point Ls to line NsPog'
14	Li-Ns Pog'	Distance from point Li to line NsPog'

### Statistical analysis

Firstly, pretreatment average value and standard error of each variable were calculated. Significant variables of the models and craniofacial structures of each group were identified by one-way analysis of variance (ANOVA) at a significance level of *P* < 0.05. Then, binary logistic regression analysis was performed and an equation was established to quantify the relationship among the significant variables and their contributions to the extraction decisions.

In order to analyze the potential error of the method during model evaluation and cephalometric tracing and measurements, 20 randomly selected models and lateral cephalometric radiographs were re-measured after an interval of 15 days. The repeatability coefficients were calculated with the analysis of variance. The coefficients were found to be very close to 1.00.

## Results

### Model studies

A summary of the model measurements and comparisons of variables among four groups were outlined in Table 4. Variables with statistical significance included molar relationship, lower anterior crowding, anterior Bolton index and anterior overjet. The maximum value of distal molar relationship was found in group 2 of 3.75 ± 0.53 mm, followed by 3.59 ± 0.53 mm in group 1, which were significantly higher than that in group 3 (0.64 ± 0.30 mm) and group 4 (1.91 ± 1.10 mm). The maximal mean value of lower anterior crowding was found in group 3 of 5.9 ± 1.06 mm, while the minimal value was found in group 1 of 1.33 ± 0.86 mm. Groups 2 and 4 were both moderately crowded. The maximal mean value of anterior overjet was 7.71 ± 1.10 mm in group 1 and anterior Bolton index was 82.18 ± 2.28% in group 2, which were significantly higher than the normal value as well as values of the other three groups. However, Spee's curve depth, overbite, and Bolton index of 6-6 showed no statistical significance among the four groups.

**Table 4:** **Mean values (standard deviation) of study cast measurements of four groups**

Variables	Group 1 (4/)	Group 2 (4/1)	Group 3 (4/4)	Group 4 (4/5)
Spee's curve depth (mm)	3.21 (0.85) a	3.13 (0.65) a	3.32 (0.64) a	3.72 (0.81) a
Overbite (mm)	3.45 (0.48) a	4.01 (0.51) a	3.99 (1.20) a	3.88(0.65) a
Overjet (mm)	7.71 (1.10) a	6.43 (0.69) b, c, d	6.25 (1.75) c, d	6.84 (0.98) d
Molar relationship (mm)	3.59 (0.53) a	3.75 (0.42) a	0.64 (0.30) b	1.92 (1.10) c
Lower anterior crowding (mm)	1.33 (0.86) a	3.60 (0.87) b, d	5.90 (1.06) c	4.03 (0.81) d
Bolton index 3-3 (%)	79.19 (2.36) a	82.18 (2.28) b	79.14 (2.29) a	79.25 (2.30) a
Bolton index 6-6 (%)	91.91 (2.29) a	92.07 (2.19) a	91.90 (2.20) a	91.92 (2.16) a

Mean values represented with different lowercase letters within a row are significantly different according to one way ANOVA (*P* < 0.05).

### Lateral radiograph analysis

The results obtained from the preliminary analysis of lateral radiographs and comparisons of each variable among four groups were presented in Table 5. Variables including ANS-Xi-Pm, NBa-PtGn, U1-NPog, L1-NPog, L1-Apog and Li-NsPog' revealed statistical significance. The ANS-Xi-Pm value of groups1 and 2 were lower than the normal value of 47°, as well as those of groups 3 and 4. The NBa-PtGn value of both groups 3 and 4 were significantly lower than normal value of 90°, with the lowest value in group 3 at 79°. Therefore, the values of ANS-Xi-Pm and NBa-PtGn indicated the horizontal growth tendency in cases of groups 1 and 2, while vertical growth tendency was more evident in group 3 and 4. In addition, L1-NPog, L1-Apog and Li-NsPog', indicating the prominence of lower incisors and lower lips, were significantly higher in group 3 and group 4, with the highest value in group 3 and the lowest value in group 1. Furthermore, the largest value of U1-NPog which reflects the prominence of upper incisors was in group 1, followed by group 3 and group 4. This was consistent with the results of overjet measured from models. However, there was no statistical significance found in angles of SNA, SNB, ANB, and SN-GoMe as well as distances of U6-PTV and Ls-NsPog'.

**Table 5:** **Mean values (standard deviation) of radiograph measurements of four groups**

Variables	Group 1 (4/)	Group 2 (4/1)	Group 3 (4/4)	Group 4 (4/5)
SNA (°)	81.57 (3.52) a	84.70 (1.85) a	82.11 (3.77) a	81.00 (3.64) a
SNB (°)	76.04 (3.06) a	79.73 (2.43) a	76.47 (3.53) a	75.44 (3.35) a
ANB (°)	5.73 (2.69) a	4.67 (0.17) a	5.64 (0.93) a	5.56 (1.78) a
SN-GoMe (°)	66.76 (4.37) a	67.40 (2.92) a	65.66 (4.59) a	65.48 (3.35) a
NBa-PtGn (°)	86.78 (4.13) a	87.47 (3.95) a	79.41 (4.74) b, c	80.79 (3.63) c
ANS-Xi-Pm (°)	45.96 (4.91) a	46.32 (5.99) a	53.00 (6.70) b, c	50.85 (4.54) c
U6-PTV (mm)	10.71 (4.66) a	12.65 (1.46) a	11.13 (4.54) a	11.60 (6.18) a
A-NPog (mm)	3.57 (3.68) a	3.91 (3.49) a	4.54 (2.02) a	4.61 (3.05) a
U1-NPog (mm)	18.05 (5.42) a	11.12 (1.92) b, d	14.89 (3.53) c	13.45 (3.38) d
L1-NPog (mm)	4.98 (2.59) a	5.92 (2.34) a, c	10.04 (3.14) b	7.83 (2.43) c
L1-APog (mm)	3.01 (2.18) a	4.75 (2.09) a, b	7.57 (3.44) b	5.80 (3.30) b, c
Ls-NsPog' (mm)	2.98 (1.36) a	2.16 (1.86) a, b	2.10 (1.79) b	2.40 (2.35) b, c
Li-NsPog' (mm)	1.45 (1.22) a	1.92 (1.81) a	4.31 (2.50) a	3.55 (2.41) a

Mean values represented with different lowercase letters within a row are significantly different according to one way ANOVA (*P* < 0.05).

### Regression analysis

For variables showing statistical significance, binary logistic regression analysis was carried out, and a regression equation was established as *Y* = 329.74-47.55X1-22.99X2-11.09X3, (*Y*, treatment outcomes of molar relationship, namely, class I or complete class II; X1, lower anterior crowding; X2, molar relationship, X3, facial growth pattern). According to the regression equation, the lower anterior crowding was the most relevant influential factor, followed by molar relationship and growth pattern. There was no significant correlation evidence with other variables investigated in our study.

## Discussion

This study was retrospectively designed to provide the possible scientific evidence and criteria for extraction decisions in Angle's class II division 1 malocclusions by comparing the initial morphologic characteristics of subjects involving four different kinds of extraction strategies, and analyzing the correlation of these factors. In spite of different extraction strategies, the outcomes in finished cases with these four different approaches had to be comparable. The six keys to normal occlusion described by Andrews are one of the most important goals and guidelines for our measure of the static relationship of successful orthodontic treatment [[16]]. All cases selected in our study achieved good facial esthetics and occlusal outcomes, which reflected that our treatment strategies were successful and effective. Undoubtedly, all patients treated in our study could be treated in other ways. But we intended to introduce the general extraction strategies for Angle's class II division 1 malocclusions with the most commonly used mechanics and the principle of simplicity in orthodontic treatment based on cases diagnosed and treated by an experienced practitioner.

In this study, we found that crowding of the lower anterior teeth, molar relationship, growth pattern, overjet, anterior Bolton index, and protrusion of the lower lip and lower anterior teeth were statistically significant factors for different extraction decisions. As suggested by the regression equation, the extraction decisions in Angle's class II division 1 malocclusions are mainly influenced, at least in part, by three variables: lower anterior crowding, molar relationship, and facial growth pattern. It also indicated that lower anterior crowding is the most relevant factor influencing the extraction decisions, followed by molar relationship and facial growth pattern. This is consistent with the findings of Nelson [[17]], who found that the correction of Angle's class II division 1 malocclusion was mainly manifested in the changes of dentition, and then the vertical changes. The study of Al-Nimri [[18]] also concluded that the decision of extraction of first or second premolars in mandible was influenced by the crowding of mandibular arch, the maxillary-mandibular plane angle, and the ratio between anterior and posterior facial heights, which are partially agreed with our results.

As the most influential factor, according to our study, the pretreatment lower anterior crowding was significantly smaller in groups 1 and 4 than those in groups 2 and 3. On the contrary, the pretreatment distal molar relationships of groups 1 and 4 were larger than those of groups 2 and 3. That is, the greater the crowding, the less the degree of distal molar relationship. This is probably because that the crowding of lower anterior teeth leads to the forward movement of posterior teeth, which impairs the distal molar relationship. The significant difference in the lower anterior crowding could be explained by the fact that first premolars are extracted to release the severe crowding, whereas second premolars are extracted when the crowding is not severe to correct the class II molar relationship [[13]]. It was suggested that the greater the mandibular crowding, the greater the tendency for a four-premolar-extraction strategy [[19]]. Besides, our results also showed that the pretreatment distal molar relationship of groups 1 and 2, in which only maxillary premolar extraction was performed and complete class II molar relationship and class I canine relationship were obtained, were significantly larger than groups 3 and 4, in which bimaxillary premolar extraction were performed and class I molar and canine relationships were achieved. In case of over cusp-to-cusp distal molar relationship, treatment of class II malocclusion with two premolar extractions achieved better occlusal success rate and greater treatment efficiency than treatment with four premolar extractions [[19]–[21]]. It is also noticeable that in group 2, which presented the distal molar relationship of cusp-to-cusp and moderate lower anterior crowding as well as the increased anterior Bolton index, a mandibular incisor was extracted with finishing complete class II molar relationship and class I canine relationship.

In growth pattern analysis, NBa-PtGn values were significantly larger in groups 1 and 2 than those in groups 3 and 4. Conversely, ANS-Xi-Pm values were significantly smaller in groups 1 and 2than those in groups 3 and 4. These indicate that the underlying growth pattern is an important factor for the extraction strategy, and this is consistent with the findings of Shearn and Woods [[22]], who believed that the growth pattern influences the madibular premolar extraction decision. Another study revealed that the growth pattern had little effect on maxillary premolar extraction decision [[23]]. In the present study, groups 1 and 2 showed horizontal growth pattern while groups 3 and 4 showed vertical growth pattern. Thus, maxillary premolar extraction only was indicated in patients with horizontal growth pattern, while bimaxillary-premolar-extraction was suitable in patients with average or vertical growth pattern. What is more, previous studies illustrated that extraction treatment was performed more likely in hyperdivergent facial type cases, whereas nonextraction treatment was more frequently carried out in mesiodivergent cases [[24]]. Schudy [[25]] also claimed that extraction of the teeth contributed to ‘closedown the bite.’ Such treatment philosophy was advocated by Sassouni and Nanda [[26]], although other studies showed that there was no significant vertical changes between either extraction treatment and nonextraction treatment, or the extraction of first premolar and second premolar [[13],[27]]. However, a recent study concluded more important factors, such as neuromuscular balance and function, beyond the extraction probably accounted for the changes of the vertical skeletal pattern [[28]].

Besides the three main factors illustrated above, specific factors should also be taken into consideration for each extraction pattern. Firstly, anterior overjet has been well acknowledged to be an important factor influencing the extraction decision. For some patients with significant overjet, extraction of upper premolars is often chosen as an alternative to orthognathic surgery [[29]]. In cases with great overjet and a good or potentially good mandibular arch, extractions can be limited to the maxillary arch only [[30]]. Shean and Woods also believed that incisor overjet acted as a main factor influencing the extraction choice of the lower arch [[22]]. In our study, the largest incisor overjet of group 1 showed great significance among the other three groups, while group 3 showed the smallest anterior overjet. Conversely, values of L1-APog and L1-NPog, which implied the degrees of lower anterior teeth protrusion, were the smallest in group 1 while the largest in group 3. Moreover, it has been reported that the lower lip position and shape are determined by the mandibular incisor position [[31],[32]]. Some others claimed that the horizontal position of the lower lip followed mandibular incisors while the vertical lip positions could be primarily directed by the maxillary incisor tip but not the mandibular incisors [[33]]. In the present study, the value of Li-NsPog', which indicates the lower lip prominence, was significantly larger in groups 3 and 4 than those in groups 1 and 2, in which premolars were extracted only in the maxilla. However, the value of Ls-NsPog', which stands for the upper lip prominence, showed no difference in our research. This probably attributed to the complicated proximal anatomical structures as well as the function of upper lip [[34]]. In addition, mesiodistal crown diameters of the upper and lower teeth in both arches should correspond for an optimal occlusion [[35]]. If a tooth size discrepancy exists, the treatment alternatives should include compensating procedures [[36]]. In the present study, the anterior Bolton ratio was significantly larger than normal in group 2, in which a mandibular incisor was extracted to obtain a complete class II molar relationship and good anterior overjet and labialingual inclination of lower incisors. Besides, the compensatory mesiolabial inclination of lower anterior teeth before or after alignment would virtually increase the total mesiodistal tooth size of mandible in a majority of class II division 1 cases. Given these, the extraction of one lower incisor in group 2 would be a desirable choice to harmonize maxillary and mandibular tooth mass, obtain proper anteroposterior position of the mandibular incisors, allow more upper anterior teeth retraction and maintain long-term stability [[37]].

## Conclusions

Angle's class II malocclusions exhibit various morphological characteristics. Extraction treatment of Angle's class II malocclusions is influenced by a number of factors rather than a single factor. Orthodontists should comprehensively take the reciprocal impact of multiple factors in to account when making an extraction decision. Based on the results of this study, the following conclusions might be drawn:

1. Crowding of the lower anterior teeth, molar relationship, and growth pattern are the three most relevant influential factors with a declining impact contributing to the extraction decisions.
2. Extractions of only two maxillary premolars could be suggested for cases presenting severe distal molar relationship over cusp to cusp, horizontal growth pattern, mild crowding of the lower anterior teeth as well as large anterior overjet more than 7 mm. Furthermore, extra extraction of one lower incisor should also be considered if the anterior Bolton index were significantly greater than normal.
3. Extractions of four first premolars could be suggested for cases with severe crowding of the lower anterior teeth, mild distal molar relationship, vertical growth pattern as well as significant lower lip prominence, whereas extractions of two maxillary first premolars and two mandibular second premolars are indicated for patients with moderate crowding of lower anterior teeth, moderate distal molar relationship less than cusp to cusp, average growth pattern, and less lower lip prominence.

## Authors’ original submitted files for images

Below are the links to the authors’ original submitted files for images. Authors’ original file for figure 1
