# Supplementary figures and images for: Morphological characteristics influencing the orthodontic extraction strategies for Angle's class II division 1 malocclusions
Source: Prog Orthod. 2014 Jul 9;15:44. doi: 10.1186/s40510-014-0044-y (PMC4884047; doi:10.1186/s40510-014-0044-y)

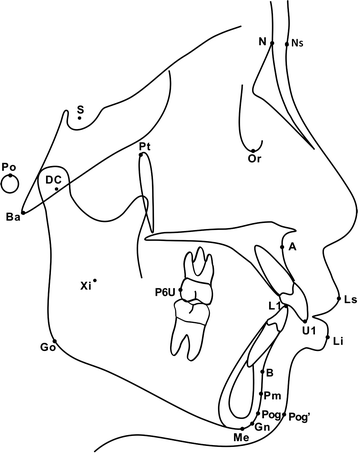

Supplement: Supplementary file 1 — Authors’ original file for figure 1 [file 40510_2014_44_MOESM1_ESM.gif]
